# Well-Differentiated Liposarcoma of the Hypopharynx Exhibiting Myxoid Liposarcoma-like Morphology with *MDM2* and *DDIT3* Co-Amplification

**DOI:** 10.1007/s12105-021-01341-5

**Published:** 2021-06-04

**Authors:** Khaled A. Murshed, Hayan Abo Samra, Adham Ammar

**Affiliations:** Department of Laboratory Medicine and Pathology, Anatomic Pathology Division, Hamad Medical Corporation, Doha, Qatar

**Keywords:** Hypopharynx, Soft tissue, Well-differentiated liposarcoma, Myxoid liposarcoma, *MDM2*, *DDIT3*

## Abstract

Well-differentiated liposarcoma (WDL) is one of the most common soft tissue sarcomas in adults. It has a predilection for middle-aged males and arises in deep-seated locations such as retroperitoneum, mediastinum, and spermatic cord. Its occurrence in young individuals at the hypopharyngeal region is an exceedingly rare event. Myxoid liposarcoma (ML)-like changes can seldom occur in some cases of WDL, which makes the diagnosis of WDL more challenging. Amplification of *DDIT3* gene in a subset of cases of WDL has shown to be associated with such unique morphology. Herein, we present a case of a 36-year-old gentleman who presented with difficulty in breathing and swallowing for 3 months duration. CT scan of the neck revealed a lesion along the posterior wall of the hypopharynx measuring 3.5 cm. Histopathologic examination revealed a tumor composed of lobules of oval to spindle cells in a prominent myxoid stroma with delicate chicken-wire vasculature. In the vicinity, there were lobules composed of variably sized adipocytes separated by thick fibrous septa that contains atypical hyperchromatic spindle cells. By immunohistochemistry, the tumor cells in both components were immunoreactive for CDK4, but negative for MDM2. Fluorescence in-situ hybridization (FISH) confirmed the presence of *MDM2* gene amplification. There was no evidence of *FUS-DDIT3* gene rearrangement, however, *DDIT3* gene was also amplified. The diagnosis of well-differentiated liposarcoma with prominent myxoid stroma was rendered. This is the first documentation of WDL with ML-like morphology harboring co-amplification of *MDM2* and *DDIT3* in the hypopharynx.

## Introduction

Liposarcomas are the most common type of sarcomas in adults [1–3]. They are divided into four main subtypes: atypical lipomatous tumor/well-differentiated liposarcoma (WDL), myxoid liposarcoma (ML), pleomorphic liposarcoma and dedifferentiated liposarcoma (DDL). ATL/WDL has predilection for middle-aged adults in the fourth and fifth decade [1–3]. Its occurrence in individuals younger than 40 years-old is uncommon [3]. ATL is the preferred terminology for tumors that arise at sites amenable to surgical resection such as the extremities and trunk, though WDL is the terminology used for tumors at deep-seated locations such as the retroperitoneum and mediastinum [1–3]. Hypopharyngeal/laryngeal liposarcomas are exceedingly rare [4–10]. Most reported cases are of well-differentiated or de-differentiated types [4–10]. Occasionally, some liposarcomas show combined morphologic features of two or more subtypes [1, 2]. In such cases, cytogenetic testing is the gold standard method for categorization of the tumor into one of these main subtypes. WDL is characterized by amplification of q13-15 region of chromosome 12, whereas ML is characterized mainly by *FUS-DDIT3* gene fusion [11–13]. Herein, we present a case of liposarcoma arising in the hypopharynx that showed combined morphologic features of WDL and ML in a 36-year-old man. The diagnosis was reached by performing cytogenetics studies. The tumor showed *MDM2* gene amplification, and eventually was categorized as well-differentiated liposarcoma. Interestingly, *DDIT3* gene amplification was also detected. To the best of our knowledge, this is the first case of hypopharyngeal WDL that exhibits ML-like morphology with *MDM2* and *DDIT3* co-amplification.

## Case Presentation

### Clinical Findings

**Fig. 1:**
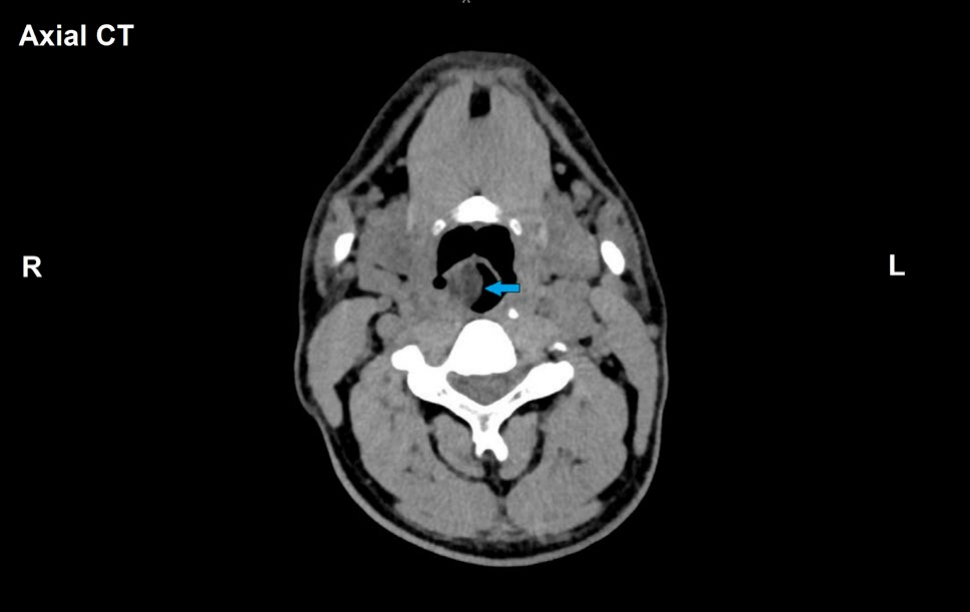
Radiological features of the hypopharyngeal well-differentiated liposarcoma with prominent myxoid stroma. Axial section CT-scan of the neck demonstrates well-defined lesion with clear fluid density contents along the posterior wall of the hypopharynx, encroaching upon the hypopharyngeal airway (blue arrow) (Color figure online)

A 36-year-old gentleman presented to the outpatient clinic with difficulty of breathing and swallowing for 3 months duration. Fiberoptic examination revealed a smooth mobile swelling arising from the right hypopharyngeal wall protruding into the larynx and causing partial airway obstruction. Computed Tomography (CT) scan of the neck revealed a 3.5 cm well-defined cystic-like lesion with clear fluid density contents along the posterior wall of the hypopharynx, encroaching upon the hypopharyngeal airway and right piriform fossa, with extension into the supra-laryngeal space (Fig. 1). It showed no enhancement on post-contrast series. The overall radiologic features were favoring a benign neoplasm/lesion, and the differential diagnosis included fibroepithelial and lymphangiomatous polyp. Hypopharyngeoscopy with mass excision was carried out.

### Histopathology and Immunohistochemistry

**Fig. 2:**
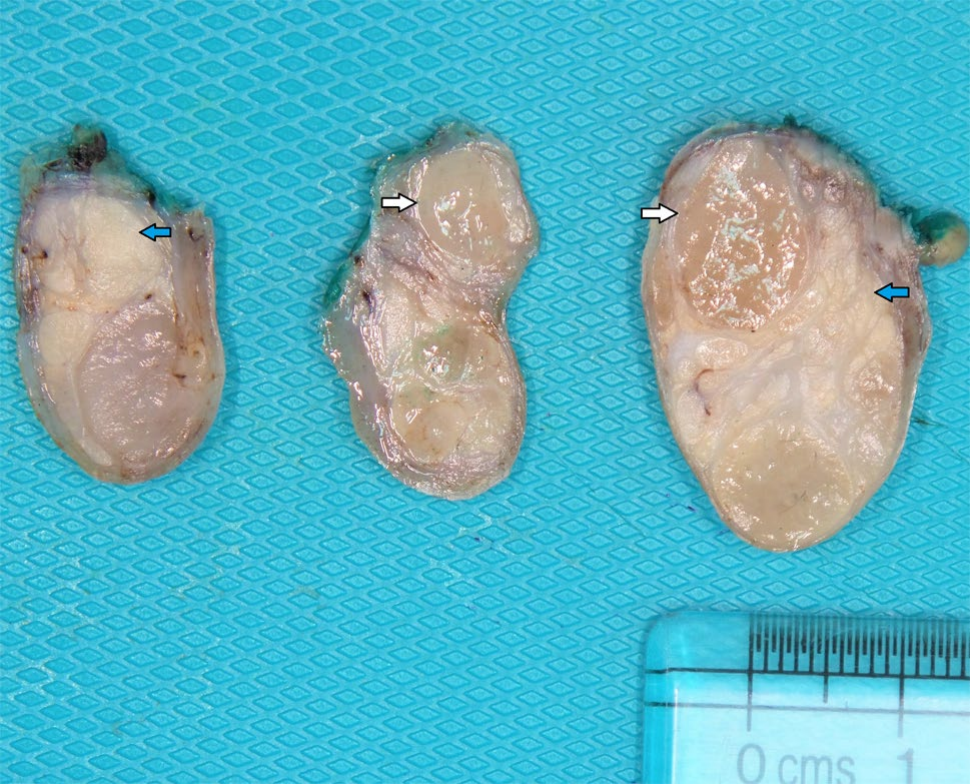
Macroscopic features of the hypopharyngeal well-differentiated liposarcoma with prominent myxoid stroma. Cut sections show lobulated tan to yellow lobulated surfaces; some areas are shiny and gelatinous (white arrows), whereas other areas are dull and rubbery (blue arrows) (Color figure online)

**Fig. 3:**
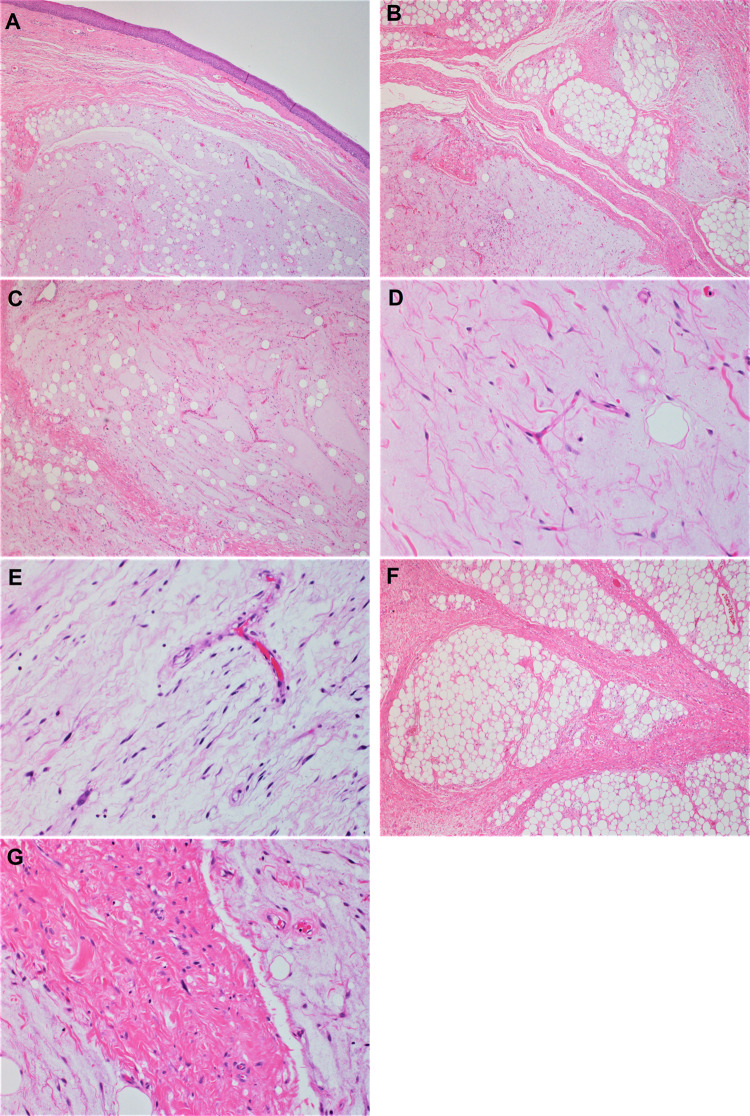
Histologic features of the hypopharyngeal well-differentiated liposarcoma with myxoid liposarcoma-like morphology (hematoxylin & eosin stain). **A**, submucosal lobules of tumor exhibiting adipocytic differentiation with overlying unremarkable squamous mucosa (H&E stain × 40). **B**, photomicrograph shows typical morphology of well-differentiated liposarcoma (upper right) with sharp distinction from the myxoid liposarcoma-like areas (lower left) (H&E stain × 100). **C**, the myxoid liposarcoma-like areas are predominantly composed of non-lipogenic spindle cells with scant adipocytic component (H&E stain × 100). **D**, High power view shows arborizing chickenwire vasculature in the myxoid liposarcoma-like areas (H&E stain × 200). **E**, Atypical hyperchromatic spindle cells within the myxoid areas (H&E stain × 200). **F**, adjacent areas show typical morphology of well-differentiated liposarcoma, there are thick fibrous bands traversing variably sized adipocytes (H&E stain × 100). **G**, atypical hyperchromatic spindle cells can be appreciated in the thick fibrous bands (H&E stain × 200)

**Fig. 4:**
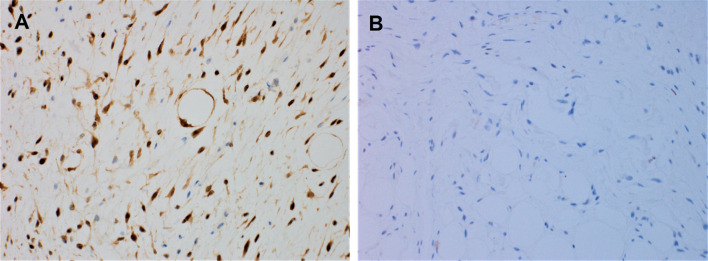
Immunohistochemical features of the hypopharyngeal well-differentiated liposarcoma. **A**, the adipocytic and atypical spindle cell component demonstrate strong and diffuse nuclear reactivity for CDK4 (× 200). **B**, they are negative for MDM2 protein (× 200)

Macroscopic examination of the resected specimen revealed a pedunculated rubbery nodule measuring 3.5 cm in maximum dimension. Cut sections showed lobulated dull rubbery and focally shiny gelatinous surfaces (Fig. 2). Microscopic examination showed squamous mucosa with underlying submucosal tumor composed of lobules of atypical oval to spindle non-lipogenic cells in a prominent myxoid stroma with arborizing chicken-wire vasculature (Figs. 3A-E). In the vicinity, there were lobules of adipose tissue separated by thick fibrous septa containing atypical hyperchromatic spindle cells (Figs. 3F-G). No definite lipoblasts were seen. There was no evidence of necrosis. Mitotic rate was 2 per 10 high-power fields (HPFs). The tumor was extending to the resection margin. Immunohistochemical studies using primary antibodies for MDM2 (clone SMP14, Zeta, dilution 1/230) and CDK4 (clone DCS-31, Zeta, dilution 1/230) revealed that the tumor cells in both components were immunoreactive for CDK4 (Fig. 4A). However, staining for MDM2 antibody was negative (Fig. 4B).

### Cytogenetics

FISH analysis was performed on 4-μm-thick sections from formalin-fixed paraffin-embedded tissue (FFPE). A representative FFPE block that contains tumor with both morphologies was selected. The testing was performed using Vysis MDM2 (12q15)/CEP12(D12Z3) FISH Probe Kit (Abbott laboratories, Illinois, U.S.A), and DDIT3 (12q13) Break Apart Probe (Mayo clinic laboratories, Minnesota, U.S.A). *MDM2* gene amplification was detected with MDM2:D12Z3 ratio of 22.3. No rearrangement of the DDIT3 probe was observed. However, amplification of DDIT3 probe region (at 12q13) was seen in all the 100 nuclei examined [100/100]. Testing for *FUS* and *EWSR1* gene rearrangement was also performed using Vysis LSI FUS Break Apart FISH Probe Kit (Abbott laboratories, Illinois, U.S.A) and Vysis LSI EWSR1 Break Apart FISH Probe Kit (Abbott laboratories, Illinois, U.S.A). Neither *FUS* nor *EWSR1* gene rearrangement were detected.

### Diagnosis and Clinical Course

Based on the immunohistochemistry and cytogenetics results, the diagnosis of well-differentiated liposarcoma with prominent myxoid stroma was rendered. The postoperative course was uneventful. Magnetic Resonance Imaging (MRI) of the neck with contrast performed 6 months post-discharge, showed no evidence of residual tumor or local recurrence. The patient will be followed up after 12 and 24 months to look for any signs of local recurrence or distant metastasis.

## Discussion

Well-differentiated liposarcoma rarely arise at the head and neck region. Its occurrence in the hypopharynx is exceedingly rare with approximately 40 cases reported in the literature [4–10]. Complete surgical resection is difficult to achieve at this site, therefore, the clinical course of hypopharyngeal WDL is typically characterized by multiple local recurrences, however, distant metastasis has not been reported [4–10]. Few cases had been described to undergo dedifferentiation after recurrence [5, 6, 10]. Hypopharyngeal WDL has predilection for middle aged males and uncommonly arise in individuals younger than 40 years [3–10]. In our case, the patient was only 36 years old, which is a rather unusual finding.

It has been shown that some cases of WDL may have prominent myxoid stroma, which makes its distinction from myxoid liposarcoma very challenging [2]. It is essential to differentiate between WDL with prominent myxoid changes from ML, as both tumors have different prognosis and treatment. ML tends to metastasize more often than WDL, unless the latter is associated with a dedifferentiated component [1, 2]. ML is more sensitive to radiotherapy and chemotherapeutic agents, whereas WDL can be curable with complete surgical resection [1, 2].

Morphologically, WDL is subdivided into adipocytic (lipoma-like) and sclerosing subtypes. Lipoma-like subtype is composed of mature adipocytes of variable sizes with scattered atypical hyperchromatic cells, whereas in the sclerosing subtype, adipocytes are separated by thick fibrous bands that contain spindle cells with atypical hyperchromatic nuclei. ML is characterized by having less adipocytic component and mainly composed of small ovoid monomorphic cells with minimal atypia, delicate arborizing capillaries “chicken-wire vasculature” and prominent mucoid stroma. In our case, some areas had ML-like morphology while other areas showed classic morphology of the sclerosing subtype of WDL. In both components, there was a degree of nuclear atypia in the tumor cells, a feature favoring the diagnosis of WDL. However, it was difficult to reach a definitive diagnosis based on histomorphology alone.

Ancillary testing by immunohistochemistry and cytogenetic studies can aid in making the distinction between both entities. *FUS-DDIT3* gene fusion, and less commonly, *EWSR1-DDIT3* are diagnostic for ML [1, 2, 13], whereas the presence of 12q13-15 amplification involving *MDM2/CDK4* genes would confirm the diagnosis of WDL [1, 2, 11, 12]. Detection of MDM2 and CDK4 protein expression can be achieved by immunohistochemistry [1, 2, 11]. In our case, the tumor cells were diffusely positive for CDK4 but negative for MDM2. We would like to emphasize here that negative staining for MDM2 does not rule out the diagnosis of WDL, as immunohistochemistry is a less sensitive method than FISH for detection of *MDM2* gene amplification [1, 2, 11, 12]. It has also been found that DDIT3 expression by immunohistochemistry can aid in the evaluation of lipomatous tumors, especially those that exhibit myxoid morphology [14]. Diffuse moderate to strong nuclear positivity for DDIT3 would support the diagnosis of ML. However, this antibody was not available in our facility. The case had been sent for FISH analysis, which confirmed the presence of *MDM2* gene amplification in our case. No *FUS* or *EWSR1* gene rearrangement were detected, and the overall findings were diagnostic of WDL.

Other diagnostic considerations in our case include atypical spindle cell/pleomorphic lipomatous tumor (ASPLT) and low-grade DDL. ASPLT is an ill-defined tumor composed of mixture of adipocytes, lipoblasts, spindle cells with mild to moderate atypia and pleomorphic multinucleated giant cells [1, 15]. The tumor is typically negative for MDM2 and CDK4 or show only focal weak staining, with consistent absence of *MDM2* and *CDK4* amplification [1, 15]. DDL is characterized by abrupt transition from WDL to non-lipogenic sarcoma, which is high-grade in most cases [1, 16, 17]. However, low-grade variants of DDL with homologous lipoblastic differentiation had been described [16]. Low-grade DDL is formed by low-grade spindle cell sarcoma with immature adipocytic component. Typically, it shows consistent diffuse nuclear reactivity for MDM2 and/or CDK4 with invariable *MDM2* amplification by FISH [1, 16]. This variant can be associated with prominent myxoid stroma. When that occurs, the distinction from WDL with ML-like morphology becomes extremely difficult. It has been found that the main distinguishing features between low-grade DDL and WDL are cellularity and mitotic rate [16, 17]. Low-grade DDL typically shows moderate cellularity and at least 5 mitoses/10 HPFs [16], while cellularity in WDL is lower and mitotic figures are rare. In our case, the cellularity was low and mitotic rate was only 2/10 HPFs, which excluded the possibility of low-grade DDL.

Interestingly, *DDIT3* gene amplification by FISH was also detected in our case. *DDIT3* gene is located on 12q13.2, a region very proximate to the *MDM2* and *CDK4* genes [18, 19]. Due to this geographic proximity, *DDIT3* gene can be amplified in a subset of cases of WDL and dedifferentiated liposarcoma. *DDIT3* encodes for a nuclear protein in the CCAAT/enhancer-binding protein (C/EBP) family, and functions as a transcription factor that plays a role in adipogenesis and adipocytic differentiation [18–21]. It has been shown in some studies that *DDIT3* gene amplification is associated with myxoid liposarcoma-like morphology [18, 19]. Mantilla et al. evaluated the presence of *DDIT3* amplification in 48 cases of de-differentiated liposarcoma and found that 33% of the cases had *DDIT3* amplification, of those, 75% were associated with ML-like morphology [18]. Therefore, *DDIT3* gene amplification may explain the presence of ML-like morphology in our case. However, the clinical and prognostic value of this finding is still not well understood.

In summary, WDL of the hypopharynx is rare and exceptional in young individuals. Some cases of WDL can have prominent myxoid stroma and features resembling ML. Immunohistochemistry and cytogenetic studies are essential to reach a definitive diagnosis in such cases. *DDIT3* gene amplification has shown to be associated with ML-like changes in few studies, which may also explain this unique morphology in our case. This the first documentation of WDL with ML-like morphology in the hypopharynx with *MDM2* and *DDIT3* co-amplification.

## Data Availability

The data that support the findings of this study are available from the corresponding author upon reasonable request.
